# Synthesis of Molecularly Imprinted Polymers for the Selective Extraction of Polymyxins from Environmental Water Samples

**DOI:** 10.3390/polym12010131

**Published:** 2020-01-06

**Authors:** Xuqin Song, Esther Turiel, Limin He, Antonio Martín-Esteban

**Affiliations:** 1 National Reference Laboratory of Veterinary Drug Residues (SCAU), College of Veterinary Medicine, South China Agricultural University, Guangzhou 510642, China; song1991yi@163.com; 2 Departamento de Medio Ambiente y Agronomía, INIA, Carretera de A Coruña km 7.5, 28040 Madrid, Spain; turiel@inia.es

**Keywords:** molecularly imprinted solid-phase extraction, polymyxins, environmental water, high-performance liquid chromatography-ultraviolet detector

## Abstract

The emergence of colistin resistance gen has aroused public concern. It is significant to assess the concentrations of polymyxins residues in aquatic environment since resistant bacteria carrying colistin resistance gen are frequently isolated from wastewater; surface water and ground water. However; no literature on the determination of polymyxins in water is available; probably due to the absence of an efficient extraction method. Accordingly; molecularly imprinted polymers were synthesized by precipitation polymerization with colistin as the template. The polymers were successfully used as sorbents for the determination of polymyxins from water based on molecularly imprinted solid-phase extraction and high-performance liquid chromatography–ultraviolet detection. The molecularly imprinted cartridge showed excellent affinity and cross-reactivity to analytes in aqueous media. Recoveries obtained from water samples were between 65.9% and 90.1%, with relative standard deviations lower than 10.2%. Limits of detection were between 1.0 and 2.0 μg L^−1^ concentration levels. Compared with C_18_ cartridge; the molecularly imprinted cartridge could remove more interference from co-extracted matrices. This method is practical for the routine monitoring of polymyxin residues in environmental water; which will benefit studies on drug-resistance and occurrence of polymyxins in the environment.

## 1. Introduction

Polymyxins have large molecular masses (>1000 Da) and unique structures of a cyclic heptapeptide and a linear tripeptide linked to a fatty acid chain through an amido bond. The two main compounds of the polymyxin family, polymyxin B (PMB) and polymyxin E (known as colistin (CS)) have strong activity against gram-negative as well as multidrug-resistant microorganisms (MDR), or the so-called “last resort” drugs [1,2]. They have a very long application history in veterinary medicine for treating and preventing bacterial infections such as bovine mastitis [3] and diarrhea [4]. Due to easy access, they are extensively used as animal feed additives in intensive husbandry systems [5]. It has been reported that the use of polymyxins (mainly CS) accounted for one-third of antibiotics in pigs and a higher ratio in poultry [6]. However, recent evidence suggested that the increasingly wide spread of CS resistance gene (MCR-1) was attributed to the excessive consumption of polymyxins by food producing animals [7]. Once most contact is made with drug resistant bacteria through the consumption of contaminated crops, infection treatment will be more difficult because of the escape of these bacteria from antibiotics [8]. Astonishingly, polymyxin resistance genes have been frequently detected in bacteria isolated from wastewater [9], surface water [10] and ground water [11]. At this regard, the Ministry of Agriculture (MOA) of China announced prohibition of CS as animal growth promoter in 2016 [12] and European Medicines Agency launched the assessment of their guidance on CS application in Europe [13].

Since pharmaceutical concentration in water is very low, efficient enrichment strategies are essential to determine trace analytes [14,15]. Solid-phase extraction (SPE) technique has been deemed as the most suitable alternative for the analysis of water samples. To date, SPE has not been used to concentrate polymyxins from water, yet, several reports introduced HLB, WCX and C_18_ sorbents to extract polymyxins from animal foodstuff [16,17], feed [18,19] and plasma [20]. Unfortunately, traditional SPE sorbents are not selective and thus result in strong matrix effects [2,17,21]. To address these problems, the use of molecularly imprinted polymers (MIPs) as sorbent material appears as an optimum alternative.

As tailor-made materials, MIPs have binding sites which can well match the template, allowing to capture target analytes selectively. Thanks to its high selectivity and mechanical robustness, molecularly imprinted SPE (MISPE) has been used in enriching trace analytes from water [22,23]. Traditionally Imprinted polymers demonstrated that hydrophobic solvents could greatly contribute to molecular recognition due to the electrostatic interactions, whereas polar solvents could generate negative effect [24]. Currently, significant attempts have been made to improve binding capacity of MIPs towards some antibiotics which are large molecular mass and high water-solubility. A typical example is the synthesis of MIPs for aminoglycoside drugs using the mixture of water and methanol (MeOH) as a porogen agent [25]. Another approach is to improve the recognition of kanamycin-MIPs in aqueous phase based on sol-gel polymer matrix which was produced by hydrolyzing silicon derivatives [26]. Up to now, only one work described a molecularly imprinted membrane as the support material to selectively absorb teicoplanin (one member of polypeptide antibiotics family) [27]. The resulting MIPs reflected high recovery of teicoplanin in serum, while the specificity and purification ability between MIPs and non-imprinted polymers (NIPs) have not been verified yet.

The present study, for the first time, describes the synthesis of selective MIPs with colistin as the template by precipitation polymerization. The MIPs were utilized as the SPE sorbent for the extraction and cleanup of polymyxins from environmental water. The MISPE cartridge could reduce the response signal of impurities dramatically, allowing the analysis of trace polymyxins (concentration level of μg L^−1^) using high-performance liquid chromatography–ultraviolet detector (HPLC–UV). The comparison between C_18_ and MISPE cartridge has proved that the latter possessed selectivity and was suitable for the analysis of real water.

## 2. Materials and Methods

### 2.1. Reagents and Materials

Methacrylic acid (MAA), divinylbenzene (technical grade 80%, DVB-80), ethylene glycol dimethacrylate (EGDMA), 2-hydroxyethyl methacrylate (HEMA) and 2,2-azobisisobutyronitrile (AIBN) were obtained from Sigma Aldrich (Madrid, Spain). MAA was purified by reduced pressure distillation. DVB-80 was freed from impurities using a self-made column of neutral alumina (Sigma Aldrich). The stabilizers of EGDMA were eliminated by an inhibitor remover column from Sigma Aldrich. AIBN was purified by recrystallization in MeOH. HPLC grade reagents including acetonitrile (ACN), formic acid (FA) and MeOH were supplied by Scharlab (Barcelona, Spain). Analytical grade reagents including ammonia (25%) (AM), acetic acid (HOAc) and dimethyl sulfoxide (DMSO) were obtained from Panreac (Barcelona, Spain). Ultrapure water was produced by a Milli-Q water system (Molsheim, France). C_18_ SPE cartridge (500 mg, 6 mL) was supplied by Supelco Analytical (Bellefonte, PA, USA).

**Figure 1:**
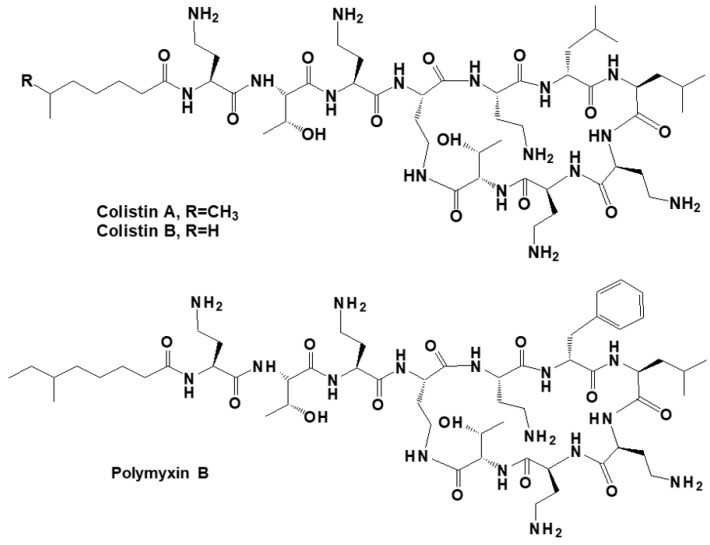
Chemical structures of colistin and polymyxin B.

Reference standards of colistin (62.9% purity) containing two major components of colistin A (CSA with the CAS number of 7722-44-3) and colistin B (CSB with the CAS number of 7239-48-7) was purchased from USP reference standards (Twinbrook Pkwy, Rockville, MD, USA) and polymyxin B (84.7% purity) was obtained from Toronto Research Chemicals (Toronto, Canada). Figure 1 presents their chemical structures. A 1 g L^−1^ concentration level of individual stock solution was prepared by weighing appropriate standard based on purity into a 10 mL of brown volumetric flask and dissolving with water, and then stored at −20 °C for no more than 3 months. Stock solutions were mixed and then diluted by fresh water daily to prepare working mixed standard solutions (100 mg L^−1^ and 10 mg L^−1^).

### 2.2. Polymer Preparation

Molecularly imprinted polymers were synthesized by precipitation polymerization. CS as the template (62.5 mg), the mixture of MAA (77.25 μL) and HEMA (25.5 μL) as the functional monomer and the mixture of DVB (135 μL) and EGDMA (690 μL) as the cross-linker were dispersed in 12.5 mL of ACN-toluene solution (75:25, *v*/*v*) in a 100 mL glass bottle, which can be sealed by a screw-cap. The above solution was sonicated for 15 min at 45 °C. Subsequently, 37.5 mL of MeOH, 40 μL of DMSO and 37.5 mg of AIBN (initiator) were added and sonicated for another 15 min. The polymerization was performed in an incubator equipped with a roller (Barloworld Scientific, Staffordshire, UK) at 60 °C and 24 rpm for 24 h. Polymers were collected by vacuum filtration and then rinsed with ACN for three times. The template removal was completed by Soxhlet extraction with 200 mL of MeOH:HOAc solution (1:1, *v*/*v*). Finally, 100 mL of MeOH was used to remove residual acid and polymers were dried at room temperature for 48 h before storage. NIPs were prepared using the same synthetic procedure with the exception that template was omitted. 100 mg of MIPs or NIPs were packed into an empty SPE cartridge (6 mL) for further experiments.

### 2.3. Characterization Techniques

An ASAP 2020 Accelerated Surface Area and Porosimetry Analyzer (Micromeritics Instrument Corporation, Norcross, GA, USA) was used to measure the porosity of polymers. The polymers (100–150 mg) were heated at 80 °C under 1025 Pa for more than 3 h before analysis. The BET method was used to calculate the specific surface areas (S), and specific pore volumes (Vp) and the average pore diameter (dp) were measured according to the BJH theory. The scanning electron micrographs (SEM) were taken from A ZEISS EVO18 microscope (Jena, Germany) at Analytical and Testing Center of South China University of Technology. A Thermo Nicolet 6700 Fourier transform infrared spectrometer (Waltham, MA, USA) was used to perform Fourier-transform infrared spectroscopy (FT-IR). The IR spectra were recorded from 500 to 4000 cm^−1^ with anhydrous KBr as the background. The thermostability of MIPs and NIPs was carried out by a NETZSCH TG 209 F1 Libra thermo gravimetric analyzer (Selb, Germany) under the following parameters: initial temperature, 40 °C; final temperature, 900 °C; heating rate, 20 °C/min.

### 2.4. Sample preparation

River, lake and spring water samples were collected from Madrid surrounding area, and then filtered through 0.45 μm nylon membrane (GE Water and process Technologies, Calgary, AB, Canada) to remove tiny particles. Collected water samples were stored in brown glass bottles at 4 °C for no more than 3 months. The MISPE cartridge was conditioned with 5 mL of MeOH and 5 mL of water, and then 100 mL of water sample was loaded. Then, 1 mL of 50% MeOH–water solution was applied to rinse the cartridge. Afterwards, 2 mL of 2% FA in MeOH was used to elute target compounds. The eluate was evaporated to dryness under nitrogen and 0.5 mL of 0.1% FA in water was used to re-dissolve the residue before HPLC analysis.

### 2.5. HPLC Analysis

HPLC analysis was preformed using an Agilent Technologies 1200 series HPLC instrument equipped with a quaternary high-pressure pump, a vacuum degasser, an auto sampler and a diode-array detector (Wilmington, DE, USA). A Phenomenex Kinetex Biphenyl column (50 mm × 4.6 mm i.d., 5 μm particle size) was used to perform chromatographic separation. The mobile phase consisted of (A) 50% ACN-water solution with 0.05% FA and (B) 0.05% FA in water. The gradient elution program was as follows: 0 min 1% A, 8 min 90% A, 8.5 min 1% A, 10 min 1% A. The flow rate was 0.75 mL min^−1^ and injection volume was 100 μL. UV monitoring was performed at 205 nm.

## 3. Results and Discussion

### 3.1. Preparation and Characterization of Imprinted Polymers

This study aimed to prepare an imprinted material which could selectivity bind CS and its structural analogues in aqueous media. The template (CS), which has amide, hydroxyl and amino groups, can form abundant hydrogen or ionic bonds with the carboxyl group of MAA. Besides, it has been reported that HEMA, as a hydrophilic monomer, could improve the hydrophilicity of the exposed surface of MIPs which will enhance specific recognition under aqueous medium [28]. Keeping this in mind, MAA and HEMA were used as co-monomers to synthesize selective MIPs.

Generally, in precipitation polymerization, polymers are formed in an appropriate solvent in which polymerization system is highly diluted, that is monomers/porogen (*w*/*v*) less than 5%. Compared with traditional bulk polymerization, imprinted particles (beads or agglomerates) prepared by this method usually hold more homogeneous binding sites, thus exhibiting excellent performance [29]. However, in order to obtain high quality polymers with stable pore structures and keep their average diameters to 1 µm, the porogenic solvent(s) should be screened according to the solubility parameter of the forming polymer network. From this point, the presence of DVB in the mixture of ACN/toluene (75/25, *v*/*v*) contributes to generate monodisperse polymer microspheres with average diameters of ~5 µm [30,31]. Accordingly, the challenge of polymer preparation was that CS is neither soluble in non-polar solvents (i.e., toluene) nor in some polar solvents (MeOH, ACN and dimethyl formamide, etc.). The key was to improve the solubility of CS in the reaction system, while circumventing the participation of water because it would significantly suppress recognition of imprinted polymers. In this case, several combinations of MeOH, ACN and toluene as porogens were preliminary screened. Practically, high proportions of ACN or toluene produced colloidal suspension of polymers on account of extremely poor solubility of CS in these porogens. Considering the solubility of CS and trying to keep mentioned porogen requirements in precipitation polymerization, mixtures of ACN, toluene and MeOH were assayed as porogen. From these experiments, it was concluded that the use of a high amount of MeOH in ACN:Toluene could effectively dissolve CS with the participation of little DMSO after sonication and finally a mixture of MeOH:ACN:Toluene (75:18.75:6.25, *v*/*v*/*v*) was chosen as optimum. However, once DVB was added as a cross-linker, it affected CS solubility and further made the polymerization mixture turbid. Increasing the amount of MeOH would negatively affect CS:MAA interactions based on hydrogen bonding and thus such porogen modification was discarded. Alternatively, mixtures of DVB and EGDMA were tested assuming that the presence of EGDMA would increase the overall polarity of the polymerization mixture and thus favoring CS solubility. After some experiments, it was concluded that a mixture of DVB:EGDMA (16/84, *v*/*v*) allowed the complete solubilization of CS in the polymerization mixture and was subjected to polymerization.

**Figure 2:**
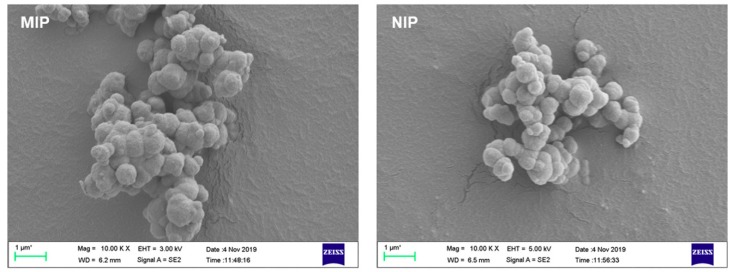
Scanning electron micrographs of MIP and NIP obtained at 10,000× magnification.

Figure 2 shows the SEM images of the MIP and NIP particles. It could be seen that agglomerates, instead of independent spherical particles, were obtained. Such agglomerates, for both MIP and NIP, were constituted by beads with particle size lower than 1 μm, which can be attributed to the great difference between the polymerization conditions used in this study and those recommended in conventional precipitation polymerization. Specific surface area of both MIP and NIP, measured by nitrogen sorption porosimetry, were as low as 10 m^2^·g^−1^, which is directly related with the low amount of toluene used in the polymerization mixture [32]. This result might limit its application in other fields where polymers with highly uniform size and shape are demanded, while this polymerization process without crushing and sieving steps has confirmed its potential application in the synthesis of imprinted polymers with high yields to be used in SPE. The IR spectra of MIPs and NIPs are given in Figure S1. MIPs and NIPs had similar adsorption bands. The typical absorption peak was at 1732 cm^−1^, the stretching vibration of C=O, resulted from the addition of EGDMA and MAA. The C=C vibration at around 1634 cm^−1^ was very weak, confirming that the polymerization reaction was successful. The thermogravimetric curves of MIPs and NIPs (Figure S2) show that both of them are quite stable within the 300 °C as their weight loss is less than 5%, indicating that they could be used in extreme temperature conditions.

### 3.2. Optimization of MISPE Conditions

The major purpose of the present paper is the application of MISPE for the determination of polymyxins in water samples. Accordingly, 1 mL of water (fortified concentration: 10 mg L^−1^ of CS) was loaded both on MIP and NIP cartridges and different washing and elution conditions were tested. Initially, it was observed that most of CS-loaded samples were neither retained by MIP nor by NIP likely due to the high initial flow-rate used (~10 mL min^−1^). By reducing the flow-rate, an increase on the retention of CS was observed although recoveries were still very low (<50%). Thus, it was decided to stop the flow during the loading step, allowing loading solution to be in contact with the polymers during different times (1–5 min). Increasing the loading time from 1 to 3 min, the recovery increased from 68.8% to 97.8%, but, no evident improvement on recovery was observed for times longer than 3 min. Hence, a loading time of 3 min was selected for further experiments. These results suggested that both the big size of CS and the low porosity of the polymers have a negative impact on mass transfer kinetics and thus CS need longer times to reach binding sites.

**Figure 3:**
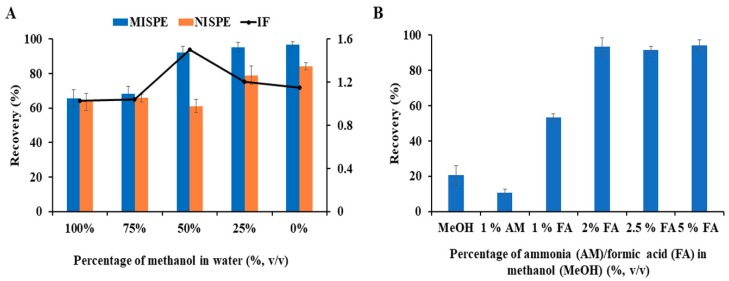
Effects of (**A**) different percentages of MeOH in water (%, *v*/*v*) as washing solutions and (**B**) methanol (MeOH), 1% ammonia (AM) in methanol and different concentrations of formic acid (FA) in methanol (%, *v*/*v*) as eluting solutions on the recovery of colistin.

Washing is a crucial step in MISPE since it has to be able to reduce non-specific interactions, as much as possible, keeping unaltered interactions inside the binding sites. Accordingly, after loading, washing solutions (1 mL) containing different amounts of MeOH in water (100%, 75%, 50%, 25% and 0%) were assayed. As presented in Figure 3A, when MeOH content is higher than 75%, large amount of CS was washed off, suggesting that interactions based on hydrogen bonding are taking place between binding sites and CS, and thus they are disrupted in the presence of a polar protic solvent (MeOH). However, for MeOH content lower than 50%, quantitative recoveries of CS in MIP cartridges were achieved. In parallel, imprinting factor (IF) (expressed as IF = RMIP/RNIP, where RMIP and RNIP are the recoveries of CS from MIP and NIP cartridges, respectively) reached a maximum value of 1.5 when a mixture of MeOH:water (50:50, *v*/*v*) was added. Therefore, 1 mL of MeOH:water (50:50, *v*/*v*) was selected as optimum washing solution since, under these conditions, non-specific interactions were minimized and satisfactory recoveries (93.7% in average) of CS on MIP was achieved.

Finally, MeOH and mixtures of AM:MeOH and FA:MeOH were tested as elution solutions. The obtained results, shown in Figure 3B, show that elution efficiencies of MeOH and AM:MeOH (1:99, *v*/*v*) were extremely achieving recoveries of CS lower than 20%. In contrast, the presence of FA improved the elution ability of MeOH, confirming, as mentioned above, that CS interactions with MIP were mainly governed by hydrogen bonding. It was clear that the highest recovery (95.1%) was achieved when the percentage of FA in MeOH was up to 2% and thus it was chosen as optimum. Then, the effect of elution volume was studied and it was observed that the recovery increased with the rising of elution volume, however, there was no significant improvement on recovery for volumes higher than 2 mL. Accordingly, 2 mL of 2% FA in MeOH was sufficient to elute CS from MIP cartridge.

### 3.3. Cross-Reactivity and Competition for the Binding Sites

**Figure 4:**
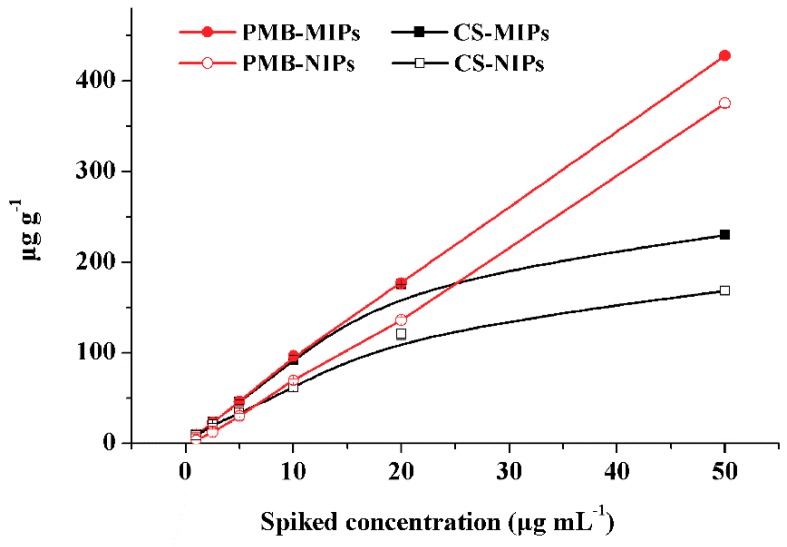
Adsorption curves of colistin (CS) and polymyxin B (PMB) on MIP and NIP.

MIPs for a specific compound uses to be able to recognize other related analytes. Accordingly, the polymers could be used to simultaneously extract CS and PMB since their chemical structures are highly similar with only one different amino acid, which has been identified as D-Leucine for CS and D-Phenylalanine for PMB [33]. Cross-reactivity was estimated by analyzing the retention of CS and PMB both in MIP and NIP. Accordingly, the independent extraction of CS and PMB under optimum conditions was studied by percolating 1 mL of standard solutions at six different concentration levels (1, 2.5, 5, 10, 20 and 50 μg mL^−1^) through both MIP and NIP cartridges. As shown in Figure 4, the amount of CS retained by MIP increases linearly reaching a plateau for concentrations higher than 20 μg mL^−1^, which correspond roughly to a maximum capacity of about 120 µg of CS per g of MIP, whereas it drops down to about 80 µg of CS per g of NIP, proving once more the presence of selective binding sites in MIP. In contrast, the amounts of PMB in MIP and NIP increased linearly within the whole range of concentration level studied. Apparently, the adsorption ability of polymers to PMB was higher than that observed for CS, suggesting that peptides skeletal structure have a remarkable effect on recognition capabilities of the polymers under study. However, since the adsorption curve of PMB on MIP had the same trend as that obtained on NIP, it might suggest that PMB retention is mainly governed by non-specific interactions. Nevertheless, recoveries of PMB on MIP were higher than those obtained on NIP, and thus PMB is able to reach specific binding sites as well. Therefore, it can be concluded that imprinted cavities in MIPs would match both CS and PMB, offering the possibility of simultaneous determination.

**Figure 5:**
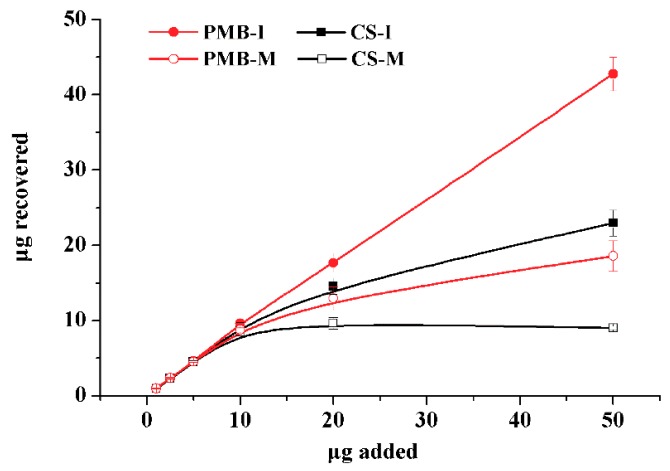
Comparison of binding curves obtained from individual colistin (CS-I), individual polymyxin B (PMB-I), and the mixture of both (CS-M and PMB-M).

Due to the limited number of binding sites in MIP, it is essential to assess whether CS or PMB showed higher binding affinity and also if they could compete each other for them. Accordingly, the competition between CS and PMB for the binding sites was investigated. Mixed standard solutions of CS and PMB at different concentrations (1~50 μg mL^−1^ in water) were subjected to the optimal MISPE process and the obtained results are depicted in Figure 5. It is clear that, at concentrations lower than 10 µg mL^−1^, simultaneous extraction of these analytes occurs, leading to linear relationships with no significant difference between the slopes obtained loading the mixture and those of loading independent analyte. However, at concentrations higher than 10 µg mL^−1^, competition for the binding sites occurs. According to the obtained results, the simultaneous extraction of CS and PMB by the proposed MISPE method from real water samples would be possible.

### 3.4. Breakthrough Volume and Reusability

The influence of the sample volume was investigated by loading different volumes of Milli-Q water (10, 20, 50, 100, 150 and 200 mL) fortified with CS and PMB at different concentrations, each one containing 10 µg of the analytes on both MIP and NIP cartridges. From Figure S3A, it is demonstrated that target compounds can be retained well on MIP cartridge (recoveries higher than 89.7%) with a maximum loading volume of 100 mL. Losses of target analytes (recovery below 66.5%) were observed for sample volume of 150 mL. In contrast, when the NIP cartridge was used (Figure S3B), the losses of analytes were found for loading volumes as low as 20 mL, demonstrating again the existence of imprinted sites on MIP and its suitability for the analysis of CS and PMB in water samples.

The reusability of MIP cartridge was evaluated by undergoing ten complete MISPE cycles (each cycle using 100 mL water spiked at 10 μg of each analyte). Between cycles, the MIP cartridge was reconditioned by washing with 10 mL of water and 10 mL of MeOH. The results showed that the retention of studied analytes decreased slightly after ten cycles, although still with recoveries higher than 82.6%. This fact indicated a good stability and durability of synthesized MIP.

### 3.5. Analytical Performance and Application of the Proposed MISPE Procedure to the Extraction of Polymyxins from Environmental Water Samples

Under optimum conditions, linearity of the whole procedure was performed by plotting matrix-matched calibration curves at six concentration levels of 3, 5, 10, 20, 30 and 50 µg/L in water samples. Good linearities were achieved in the experimental concentration range, with correlation coefficients (r) higher than 0.998. The limit of detection (LOD) and limit of quantification (LOQ), figured out through assaying the corresponding concentration at the points of signal-to-noise ratios of 3:1 and 10:1, are summarized in Table 1. LODs and LOQs were within the range of 1.0~2.0 μg L^−1^ and 3.0~6.5 μg L^−1^, respectively, depending upon the kind of sample and target analyte. The accuracy and precision of the proposed method, in terms of recovery and relative standard deviation (RSD), were measured by analyzing spiked water samples with both CS and PMB at 10 µg L^−1^ concentration level. Recoveries (also shown in Table 1) obtained for target analytes in river, lake and spring water were between 65.9% and 90.1%. Besides, the proposed method presents a satisfactory precision with intra-day and inter-day RSDs below 10.2% and 6.4%, respectively. Good recoveries and repeatability of this method could satisfy the fit-for-purpose criteria of the Commission Decision 2002/657/EC [34].

**Figure 6:**
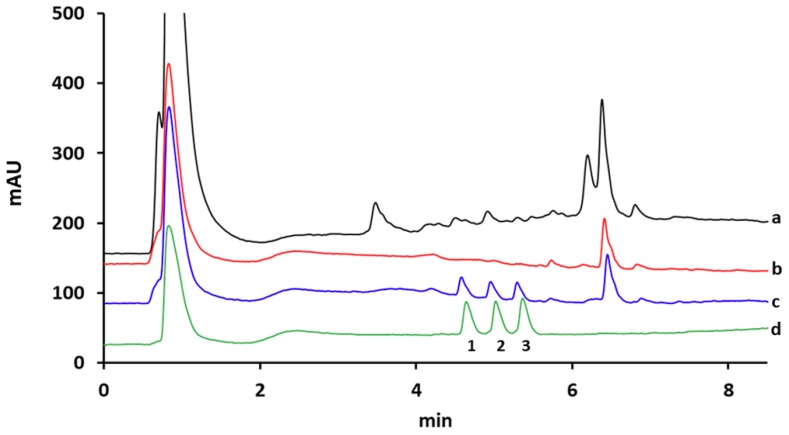
HPLC chromatograms of non-spiked river water extracted with (**a**) C_18_ cartridge and (**b**) MIP cartridge, (**c**) spiked river water at 10 μg L^−1^ concentration level extracted with MIP cartridge and (**d**) the corresponding standard solution. Peak identifications: 1, colistin B; 2, colistin A; 3 polymyxin B.

Figure 6 shows the chromatograms obtained from the blank and spiked (10 µg L^−1^) river water samples after SPE on commercial C_18_ and on proposed MIP cartridges. The C_18_ cartridge was conditioned with 5 mL of MeOH and 5 mL of water. Impurities were removed by 3 mL of 50% MeOH–water solution and, finally, the target compounds were eluted with 2 mL of 2% FA in MeOH. It is obvious that MIP cartridge (Figure 6c) was able to significantly reduce the amount of co-extracted matrix compounds impurities allowing the detection of target analytes. However, the corresponding chromatogram obtained after SPE on the C_18_ cartridge (Figure 6a) contained large amounts of unknown peaks at retention times of target analytes and presents a noisy baseline, thus hampering their accurate determination. Figure S4 shows the obtained chromatograms after MISPE of target compounds from lake and spring water samples. As can be observed, there were no interference peaks at the retention times of target analytes allowing their unequivocal detection. The obtained chromatograms confirmed the excellent performance of the synthesized MIP as SPE sorbent for the enrichment and purification of trace amounts of polymyxins in water samples from different sources.

Finally, there was no template bleeding in the development of the present work, and thus guaranteeing the accurate quantification of analytes.

## 4. Conclusions

As colistin is the last alternative to treat MDR infections, the prevalence of MCR-1 has become a critical challenge for treating and controlling infectious diseases. It is important to highlight the surveillance of polymyxin residues in aquatic environments because of the generation and spread potential of MCR-1. In the present study, an efficient and selective HPLC–UV method based on imprinted material for the determination of polymyxins from environmental water was developed. MIP synthesized by precipitation polymerization had higher affinity to target compounds than its corresponding NIP, confirming the presence of specific binding sites. Compared with a traditional C_18_ cartridge, the MIP cartridge could remove much more matrix interferences and reduce baseline noises significantly, allowing to obtain low LOD using HPLC–UV detector. This method could be applied to detect trace amounts of polymyxins in water, which will benefit the control of microbiological risk associated with the consumption of polymyxins. Moreover, in order to prevent polymyxins from being released into the aquatic environment and reduce the occurrence and dissemination of MCR-1, more measures need to be enforced including decreasing the usage of polymyxins in veterinary medicine as well as performing strict wastewater treatment processes.

## Figures and Tables

**Table 1:** Accuracy, precision, limit of detection and limit of quantification of colistin A, colistin B and polymyxin B from different kinds of water samples spiked with the mixture of target analytes (10 µg L^−1^ for each) after MISPE treatment.

Water Source	Analyte *^a^*	Intra-Day Recovery (RSD *^b^*, %, n = 3)			LOD ^*c*^	LOQ *^d^*	LOQ *^d^*
		I	II	III	(RSD, %, n = 9)	(µg L^−^^1^)	(µg L^−^^1^)
River	CSA	74.7 (3.7)	65.9 (4.5)	75.7 (3.2)	72.1 (7.3)	2.0	6.5
	CSB	77.2 (3.6)	72.1 (3.8)	72.1 (1.9)	73.8 (4.6)	1.5	5.0
	PMB	83.7 (3.2)	76.9 (9.7)	83.6 (1.4)	81.4 (6.3)	1.0	3.0
Spring	CSA	72.3 (2.1)	71.6 (4.7)	76.8 (1.2)	73.6 (4.2)	1.0	3.0
	CSB	74.6 (2.2)	81.8 (3.9)	74.4 (3.0)	76.9 (5.5)	1.0	3.0
	PMB	85.5 (5.3)	83.1 (4.3)	80.9 (10.2)	83.2 (6.4)	1.0	3.0
Lake	CSA	83.1 (3.9)	84.4 (6.2)	86.4 (9.5)	84.6 (6.3)	2.0	6.0
	CSB	85.6 (3.7)	86.7 (2.3)	85.1 (3.6)	85.8 (3.1)	1.5	5.0
	PMB	83.8 (3.1)	90.1 (1.8)	84.5 (2.3)	86.2 (4.0)	1.0	3.0

*^a^* CSA, colistin A; CSB, colistin B; PMB, polymyxin B; *^b^* RSD, relative standard deviation; *^c^* LOD, limit of detection; *^d^* LOQ, limit of quantification.

## Supplementary Materials

The following are available online at https://www.mdpi.com/2073-4360/12/1/131/s1, Figure S1: IR spectra of MIPs and NIPs, Figure S2: The thermogravimetric analysis of MIPs and NIPs, Figure S3: Effect of loading volume on the recoveries of colistin A (CSA), colistin B (CSB) and polymyxin B (PMB) obtained after SPE onto (A) MIP and (B) NIP cartridges, Figure S4: HPLC chromatograms obtained after MISPE of target analytes from lake and spring water: (a) non-spiked water, (b) spiked water at 10 μg L^−1^ concentration level and (c) mixed standard solution. Peak identifications: 1, colistin B; 2, colistin A; 3 polymyxin B.
