# Synthesis of Molecularly Imprinted Polymers for the Selective Extraction of Polymyxins from Environmental Water Samples

**DOI:** 10.3390/polym12030594

**Published:** 2020-03-08

**Authors:** Xuqin Song, Esther Turiel, Limin He, Antonio Martín-Esteban

**Affiliations:** 1 National Reference Laboratory of Veterinary Drug Residues (SCAU), College of Veterinary Medicine, South China Agricultural University, Guangzhou 510642, China; song1991yi@163.com; 2 Departamento de Medio Ambiente y Agronomía, INIA, Carretera de A Coruña km 7.5, 28040 Madrid, Spain; turiel@inia.es

The authors wish to make a change to the published paper [1]. In the original manuscript, there are mistakes in the headers of columns six and seven in Table 1. The corrected Table 1 is presented below.

The authors apologize for any inconvenience caused and the change does not affect the scientific results. The manuscript will be updated, and the original will remain online on the article webpage at https://www.mdpi.com/2073-4360/12/1/131.

## Figures and Tables

**Table 1:** Accuracy, precision, limit of detection and limit of quantification of colistin A, colistin B and polymyxin B from different kinds of water samples spiked with the mixture of target analytes (10 µg L^−1^ for each) after MISPE treatment.

Water Source	Analyte *^a^*	Intra-Day Recovery (RSD *^b^*, %, n = 3)			Inter-Day Recovery	LOD *^C^*	LOQ *^d^*
		I	II	III	(RSD, %, n = 9)	(µg L^−1^)	(µg L^−1^)
River	CSA	74.7 (3.7)	65.9 (4.5)	75.7 (3.2)	72.1 (7.3)	2.0	6.5
	CSB	77.2 (3.6)	72.1 (3.8)	72.1 (1.9)	73.8 (4.6)	1.5	5.0
	PMB	83.7 (3.2)	76.9 (9.7)	83.6 (1.4)	81.4 (6.3)	1.0	3.0
Spring	CSA	72.3 (2.1)	71.6 (4.7)	76.8 (1.2)	73.6 (4.2)	1.0	3.0
	CSB	74.6 (2.2)	81.8 (3.9)	74.4 (3.0)	76.9 (5.5)	1.0	3.0
	PMB	85.5 (5.3)	83.1 (4.3)	80.9 (10.2)	83.2 (6.4)	1.0	3.0
Lake	CSA	83.1 (3.9)	84.4 (6.2)	86.4 (9.5)	84.6 (6.3)	2.0	6.0
	CSB	85.6 (3.7)	86.7 (2.3)	85.1 (3.6)	85.8 (3.1)	1.5	5.0
	PMB	83.8 (3.1)	90.1 (1.8)	84.5 (2.3)	86.2 (4.0)	1.0	3.0

*^a^* Colistin A (CSA); colistin B (CSB); polymyxin B (PMB); *^b^* relative standard deviation (RSD); *^c^* limit of detection (LOD); *^d^* limit of quantification (LOQ).
