# Supplementary material for: Synthesis of Molecularly Imprinted Polymers for the Selective Extraction of Polymyxins from Environmental Water Samples
Source: Polymers (Basel). 2020 Jan 6;12(1):131. doi: 10.3390/polym12010131 (PMC7022724; doi:10.3390/polym12010131)
Supplement: Supplementary file 1 [file polymers-12-00131-s001.pdf]

Supplementary Materials

Synthesis of molecularly imprinted polymers for the selective extraction of polymyxins from environmental water samples

Xuqin Song [1], Antonio Martín-Esteban [2], Limin He [1,*] and Esther Turiel [2,*]

[1] National Reference Laboratory of Veterinary Drug Residues (SCAU), College of Veterinary Medicine, South China Agricultural University, Guangzhou 510642, China; song1991yi@163.com; liminokhe@scau.edu.cn

[2] Departamento de Medio Ambiente y Agronomía, INIA, Carretera de A Coruña km 7.5, 28040 Madrid, Spain; amartin@inia.es; turiel@inia.es

* Correspondence: turiel@inia.es; Tel.: + 34 91 3478700; fax: + 34 91 3474008

* Co-Correspondence: liminokhe@scau.edu.cn; Tel.: +86 20 85280665; fax: +86 20 85284896

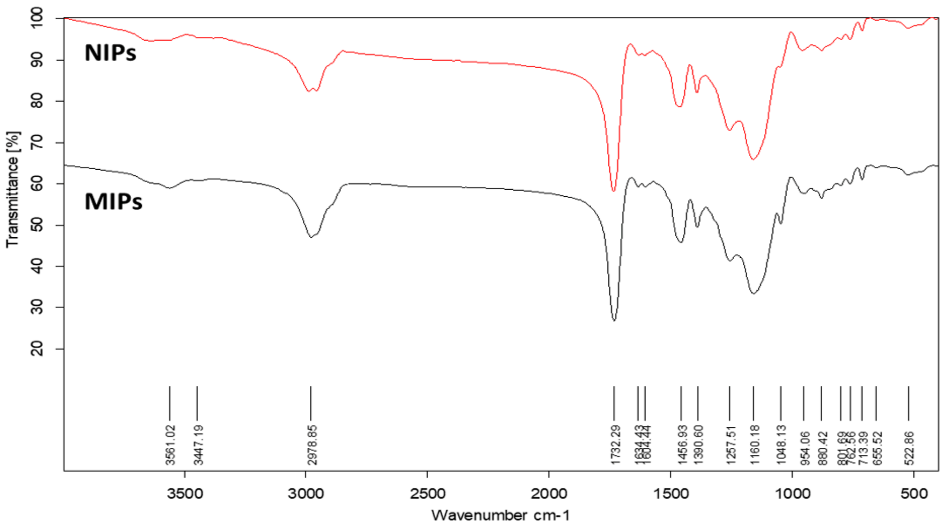

Figure S1. IR spectra of MIPs and NIPs

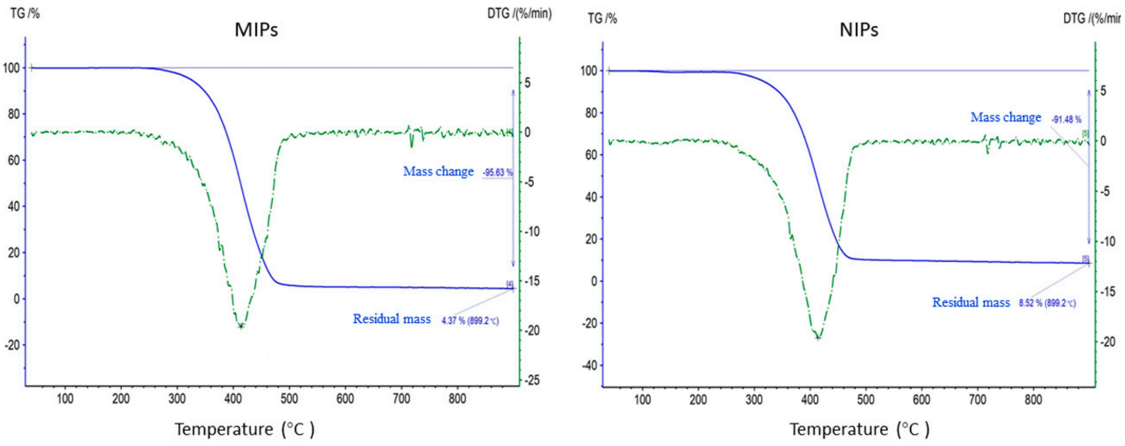

Figure S2. The thermogravimetric analysis of MIPs and NIPs

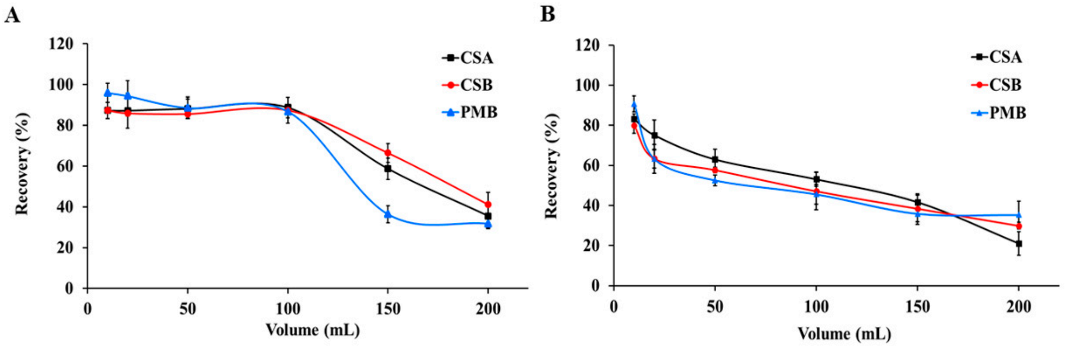

Figure S3. Effect of loading volume on the recoveries of colistin A (CSA), colistin B (CSB) and polymyxin B (PMB) obtained after SPE onto (A) MIP and (B) NIP cartridges.

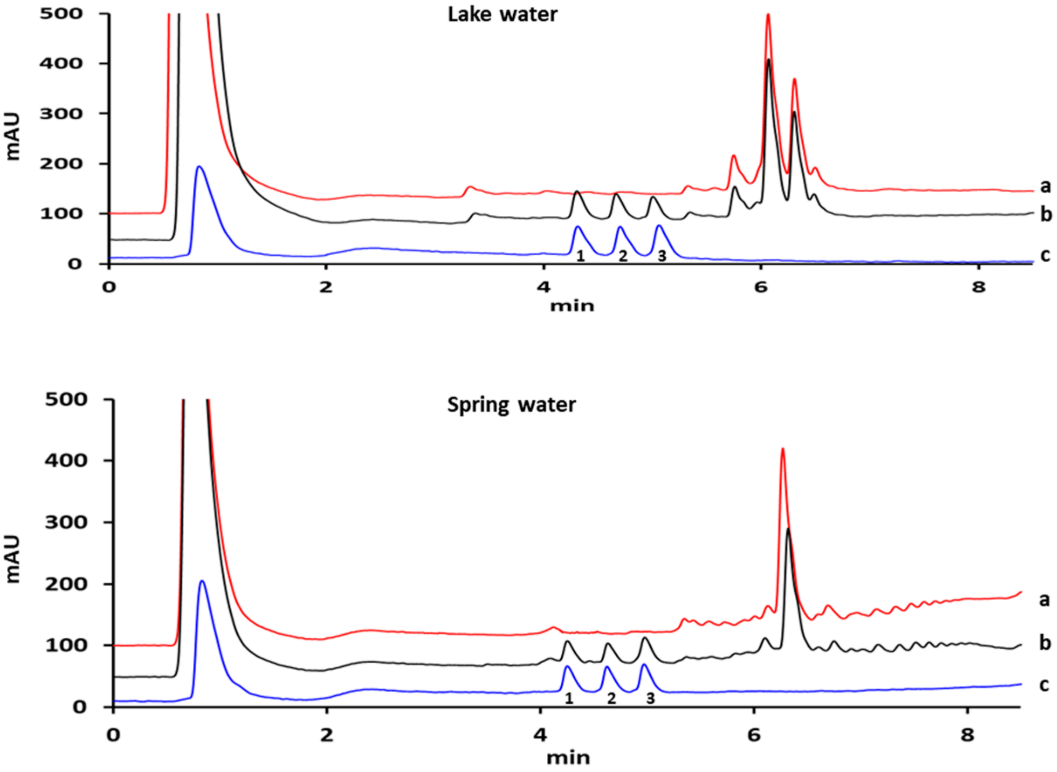

Figure S4. HPLC chromatograms obtained after MISPE of target analytes from lake and spring water: (a) non-spiked water, (b) spiked water at 10 µg L^{-1} concentration level and (c) mixed standard solution. Peak identifications: 1, colistin B; 2, colistin A; 3 polymyxin B
